# Reaction selectivity of homochiral versus heterochiral intermolecular reactions of prochiral terminal alkynes on surfaces

**DOI:** 10.1038/s41467-019-12102-y

**Published:** 2019-09-11

**Authors:** Tao Wang, Haifeng Lv, Jianmin Huang, Huan Shan, Lin Feng, Yahui Mao, Jinyi Wang, Wenzhao Zhang, Dong Han, Qian Xu, Pingwu Du, Aidi Zhao, Xiaojun Wu, Steven L. Tait, Junfa Zhu

**Affiliations:** 1 National Synchrotron Radiation Laboratory, Department of Chemical Physics and Key Laboratory of Surface and Interface Chemistry and Energy Catalysis of Anhui Higher Education Institutes, University of Science and Technology of China, 230029 Hefei, People’s Republic of China; 2 Hefei National Laboratory of Physical Sciences at the Microscale, University of Science and Technology of China, 230026 Hefei, People’s Republic of China; 3 Department of Materials Science and Engineering, CAS Key Laboratory of Materials for Energy Conversion, and CAS Center for Excellence in Nanoscience, University of Science and Technology of China, 230026 Hefei, People’s Republic of China; 4 Department of Chemistry, Indiana University, Bloomington, IN 47405 USA; 5 Dalian National Laboratory for Clean Energy, 116023 Dalian, People’s Republic of China

**Keywords:** Reaction kinetics and dynamics, Scanning probe microscopy, Density functional theory

## Abstract

Controlling selectivity between homochiral and heterochiral reaction pathways on surfaces remains a great challenge. Here, competing reactions of a prochiral alkyne on Ag(111): two-dimensional (2D) homochiral Glaser coupling and heterochiral cross-coupling with a Bergman cyclization step have been examined. We demonstrate control strategies in steering the reactions between the homochiral and heterochiral pathways by tuning the precursor substituents and the kinetic parameters, as confirmed by high-resolution scanning probe microscopy (SPM). Control experiments and density functional theory (DFT) calculations reveal that the template effect of organometallic chains obtained under specific kinetic conditions enhances Glaser coupling between homochiral molecules. In contrast, for the reaction of free monomers, the kinetically favorable reaction pathway is the cross-coupling between two heterochiral molecules (one of them involving cyclization). This work demonstrates the application of kinetic control to steer chiral organic coupling pathways at surfaces.

## Abstract (web summary)

Controlling selectivity between homochiral and heterochiral reaction pathways on surfaces is intriguing but challenging. Here, the authors demonstrate strategies in steering the reactions of prochiral terminal alkynes between the homochiral and heterochiral pathways by tuning the precursor substituents and the kinetic parameters.

## Introduction

A molecular structure is chiral if it cannot be superimposed onto its mirror image. Owing to the homochiral nature of the biochemistry of life on the earth, molecular chirality continues to be one of the most important research topics in biology and chemistry^1^. The invention of scanning tunneling microscope (SPM) has provided great opportunities to study chiral structures on solid surfaces at the atomic level^2–6^. The construction of two-dimensional (2D) chiral supramolecular self-assembly structures has been intensely studied over the past decade^7–10^, with growing interest in understanding systems of greater complexity and the mechanisms of enantiospecific reactions^11,12^. Although the overall 2D surfaces are achiral (racemic), homochiral supramolecular domains can be obtained from the adsorption of chiral species^2,5,10^ or by adsorption of prochiral precursors that adopt a chiral character when adsorbed to the surface^8,9^, as in the present case. Beyond the relatively weak supramolecular (non-covalent) self-assembly, recent developments in on-surface synthesis^13–15^ pave the way to study chemical reactions between chiral molecules to produce stable chiral products on surfaces. The confinement effect of a 2D surface will kinetically favor either homochiral or heterochiral intermolecular reactions and, as a result, the reactions typically exhibit diastereoselectivity^16–18^.

However, the selective control of homochiral vs. heterochiral intermolecular reaction pathways on surfaces remains elusive. For on-surface synthesis under ultra-high vacuum (UHV) environments, the control of a multipath reaction is much more difficult^19,20^ than in wet chemistry where different reaction selectivities can be achieved using appropriate catalysts, ligands, or substituents^21–23^.

**Fig. 1:**
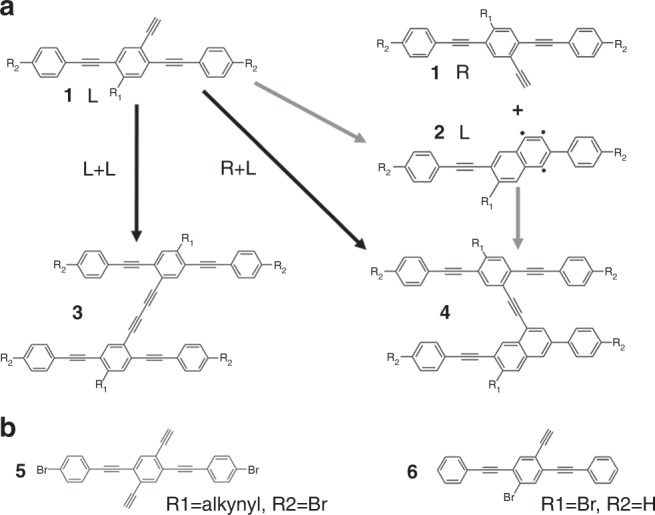
The reaction scheme of alkyne molecules. **a** The potential reaction pathways of chiral terminal alkyne **1**, forming **3** from homochiral intermolecular reaction and **4** from heterochiral intermolecular reaction. **b** The precursor molecules used in this work

On-surface reaction of alkyne has attracted increasing attention recently due to its great potential for the synthesis of graphdiyne via Glaser coupling^24,25^ and the creation of biradical phenyl via Bergman cyclization of *cis*-enediyne units^26–28^. The introduction of chirality to the alkynes for on-surface reactions is interesting for advancing understanding of alkyne reactivity at surfaces and as a model system for addressing the challenging problem of steering chirality in coupling reactions at surfaces. As shown in Fig. 1, for reactions starting from prochiral alkyne **1** (the chirality is induced by the surface confinement), the competitive pathways of Glaser coupling or Bergman cyclization could generate different products. Interestingly, the intermolecular Glaser reaction will occur only between two homochiral molecules **1** (forming **3**); in contrast, only heterochiral coupling can happen between **1** and the Bergman cyclization product **2**, forming H-type product **4** (Supplementary Fig. 1). Note that **2** inherits the chirality of **1**. Consequently, it is possible that the homochiral vs. heterochiral intermolecular reactions of **1** give rise to the formation of different chemical species (**3** and **4**). In a previous work^29^, diacetylene bridged chiral covalent chains (product **3**) were obtained via the Glaser coupling of molecules **5** on Ag(111) by undergoing an organometallic intermediate state. The introduction of Br is supposed to weaken the C–H bond of the terminal alkyne. In that work, structure **4** appears as a minor byproduct.

Herein, we report the highly selective synthesis of H-type product **4** from heterochiral intermolecular reaction on Ag(111) by tuning the substituent groups of **1** and deliberately controlling the reaction kinetics. Molecule **6** with only one terminal alkynyl is used as the precursor. The intermediate and final structures are characterized by scanning tunneling microscopy (STM), non-contact atomic force microscopy (nc-AFM), and synchrotron radiation photoelectron spectroscopy (SRPES). A series of control experiments and DFT calculations unravel the mechanism for the distinct reaction selectivity between **5** and **6**. This work offers insight into the competition between homochiral vs. heterochiral intermolecular reactions on surfaces.

## Results

### Formation of H-type product 4

**Fig. 2:**
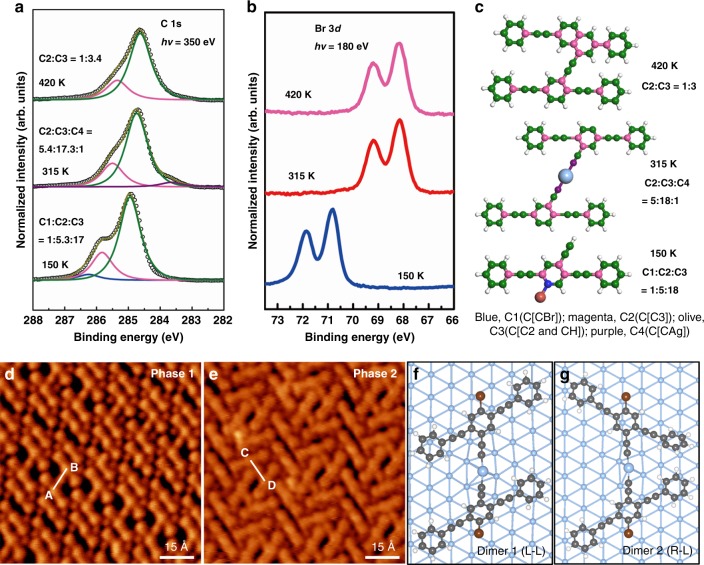
SRPES of reaction products and STM of the 315 K sample. **a**, **b** C 1*s* and Br 3*d* SRPES of the samples prepared by depositing molecule **6** on Ag(111) held at 150 K, 315 K, annealing the 315 K sample to 420 K, respectively. The photo energies for C 1*s* and Br 3*d* are 350 and 180 eV, respectively. **c** The molecular models of the main products in each temperature points. Different carbon atoms are depicted by different colors to illustrate their chemical environments. The ideal ratios of these C atoms are shown in the right. **d**, **e** STM images showing two different ordered phases of products upon deposition of **6** on Ag(111) held at 315 K. Tunneling parameters: **d** *V* = −2.1 V, *I* = −0.3 nA; **e** *V* = −1.5 V, *I* = −0.2 nA. The center-to-center of the monomer in organometallic dimers are marked by white lines AB and CD. **f**, **g** The optimized bimolecular organometallic configurations formed at 315 K. Color code: C, gray; H, white; Br, red; Ag, silver

Molecule **6** remains intact when vapor deposited onto Ag(111) at a surface temperature of 150 K, as confirmed by SRPES results (Fig. 2a, b) and consistent with previous study^29^. The Br 3*d* spectrum at 150 K exhibits a single spin–orbit split doublet (3*d*_3/2_ and 3*d*_5/2_) with Br 3*d*_5/2_ at a binding energy (BE) of 70.8 eV, indicating that the C–Br bonds are intact^30,31^. The C atoms in molecule **6** can be divided into three groups according to their different chemical environments (different BEs)^32–34^. They are named as C1 to C3, from the highest BE to the lowest (Fig. 2c): C1 is the C atom connected to the Br atom (C[CBr]); C2 has three neighboring C atoms (C[C3]); and C3 includes both the C with two neighboring C atoms (C[C2]) and the terminal C of ethynyl (C[CH]). The C 1*s* spectrum at 150 K is fitted with the three C components in a ratio of 1:5.3:17, and their binding energies are 286.2, 285.8, and 284.9 eV, respectively, in agreement with typical literature values^30,34^. This ratio matches well the value of 1:5:18 derived from the molecular structure of **6**. These intact molecules exhibit strong mobility on the Ag(111) surface at ~90 K, because they cannot be scanned stably by STM at a conventional bias voltage of tip (1–2 V). At a high bias voltage (>3 V), some molecules can be imaged (Supplementary Fig. 2).

After deposition of **6** at 0.6 monolayer (ML) onto the Ag(111) surface held at 315 K, two types of well-ordered self-assembly phases are formed (each of them accounts for 40% of organic adsorbates), as shown in Fig. 2e (overview STM images and disordered phases are shown in Supplementary Fig. 3). The two phases are each composed of both monomers and dimers with a bright dot in its center. The dimers in phase 1 exhibit oblique H shape and in phase 2 they show A shape. According to previous studies^29,35,36^, the dimeric species is an organometallic structure and the bright dot is assigned as a Ag adatom. This is supported by the SRPES result at 315 K (Fig. 2a, b). At 315 K, the dissociation of C–Br bond occurs completely, which is evidenced by the BE shift of Br 3*d*_5/2_ from 70.8 to 68.2 eV (Fig. 2b)^30^. These Br adatoms are identified as relatively dark dots in the STM images (Fig. 2d, e) surrounding the organic molecules via Br⋯H bonds. Also, the C 1*s* signal of C–Br (C1) disappears at 315 K. Meanwhile, C2 and C3 shift slightly to BE of 284.9 and 284.1 eV, respectively, and a new peak, C4, is developed at a low BE of 283.1 eV. C4 matches the fingerprint of C–metal bonds^30,37^, indicating the formation of C–Ag bonds in this system. Note that besides the alkynyl–Ag bond in the organometallic dimer, the dehydrogenated monomers may also interact with the Ag(111) surface and give contribution to the intensity of C4^31,34^, because radical alkynyl has stronger interactions with Ag(111) than radical phenyl due to the existence of a localized unpaired electron in its C terminal. The ratio of C2:C3:C4 (5.4:17.3:1) derived from the C 1*s* spectrum matches the molecular model (5:18:1) well. The observed BE shift of C2 and C3 to low BE direction from 150 to 315 K is partly attributed to the work-function increase of Ag(111) caused by the chemisorbed Br adatoms^38^. The distances between the central phenyl groups of the molecular backbones marked as AB and CD in Fig. 2d, e (AB = 12.1 ± 0.3 Å, CD = 12.3 ± 0.3 Å) imply that the organometallic dimer should be linked via alkynyl–Ag–alkynyl bonds^29^. Interestingly, the oblique H-type dimers originate from the dimerization of two homochiral molecules, while the A-type dimers are from two heterochiral molecules. The molecular densities are calculated to be 0.60 monomer per nm^2^ for phase 1 and 0.64 monomer per nm^2^ for phase 2, respectively.

To further elucidate the organometallic structures, DFT calculations were performed (Fig. 2f, g, oblique views in Supplementary Fig. 4), which confirm the stability of both the H-type and A-type dimers as energy minimized structures. In these calculations, we leave the C–Br bonds intact to avoid the slow convergence that would be caused by the unpaired electrons. For both structures, the terminal alkynyls bind with Ag adatoms, resulting in tilted configurations. In dimer 1 two molecules are parallel to each other. In dimer 2 the two molecules have an angle of about 72° and the adjacent benzene rings rotate to form a dihedral angle of about 15° to avoid the repulsive forces of hydrogen atoms. The adsorption energy of dimer 1 is 0.07 eV lower than that of dimer 2. The small energy difference implies that both dimers can be obtained simultaneously.

**Fig. 3:**
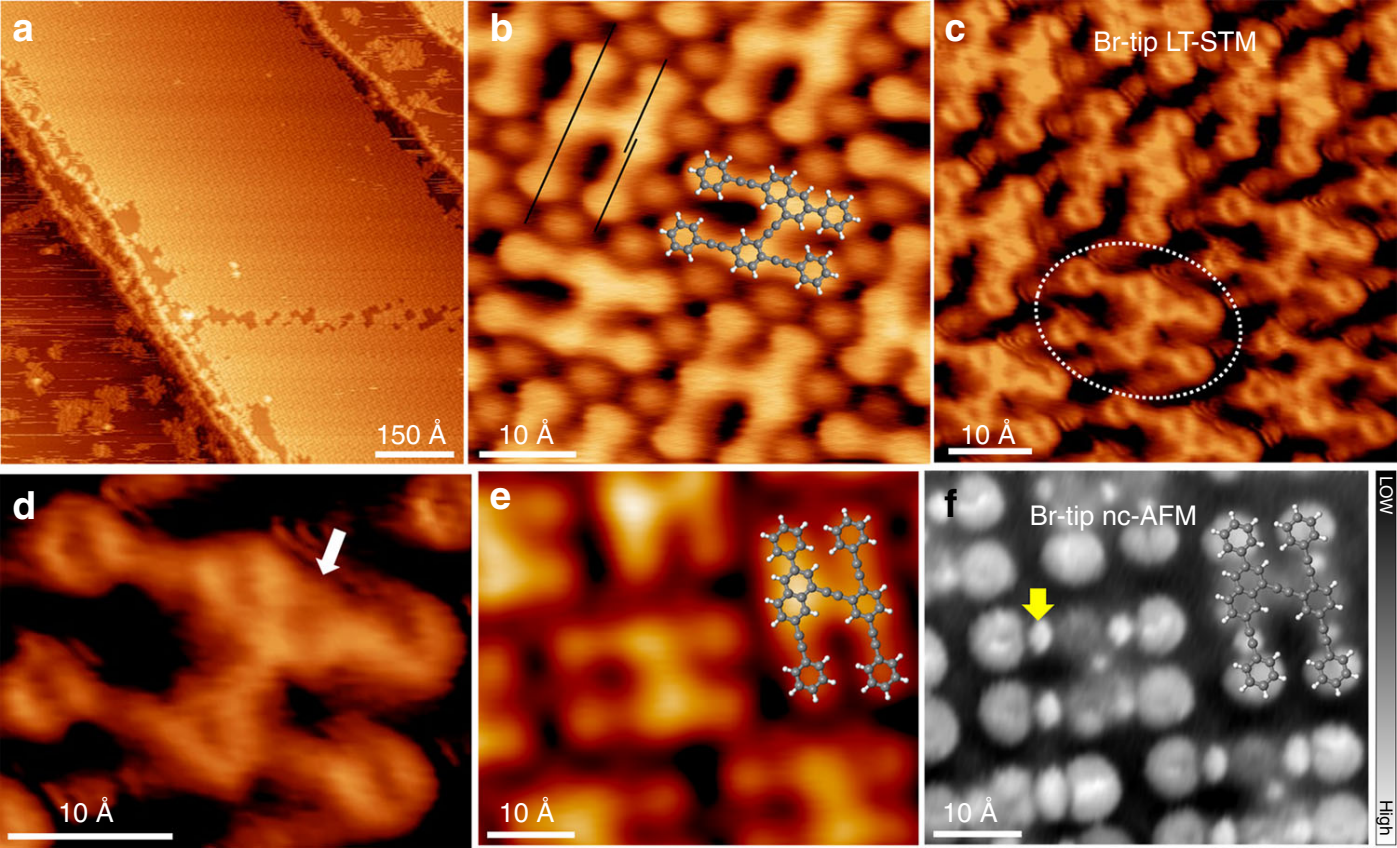
STM images and nc-AFM frequency shift maps of the H-type product **4**. **a**, **b** Overview and high-resolution STM images of the sample after annealing to 420 K, recorded at 93 K. The axes of molecular backbones are marked by black lines. **c** LT-STM image of H-type product **4** by using a Br-tip, recorded at 4.3 K. **d** Zoom-in LT-STM image of the molecule circled in **c**. The formed phenyl is pointed by the white arrow. **e**, **f** STM image and the corresponding nc-AFM frequency shift map of a same area composed by H-type product **4**, by using a Br-tip. One of the ethynylenes of **4** in nc-AFM frequency shift map is pointed by the yellow arrow. Tunneling parameters: **a** *V* = −2 V, *I* = −0.2 nA; **b** *V* = −0.2 V, *I* = −0.6 nA; **c**, **d** *V* = 0.1 V, *I* = 1.0 nA; **e** *V* = 1 V, *I* = 0.2 nA; **f** *V* = 0 V, Δ*f*: −9.8 to −4.8 Hz. Color code: C, gray; H, white

Upon annealing the sample to 420 K, it is surprising that large-area close-packed islands composed of regular H-type products are formed, as shown in Fig. 3a. For comparison, different molecular coverages before annealing are used. It shows that the H-type products dominate at every coverage (0.1, 0.3, 0.6, and 0.9 ML, Supplementary Fig. 5). Figure 3b exhibits the high-resolution STM of the H-type product. The smooth and seamless connection implies covalent linkages. In addition, it is clear that one of the two monomers in each H unit shows non-axis character (marked as black lines); thus, an intramolecular cyclization may be involved in its structure. In other words, the product **4** from reaction between two heterochiral monomers (R1, R2 = H, Fig. 1) is probably obtained. This is strongly supported by the fact that the molecular model of **4** shows an excellent match with the STM morphology. A more direct evidence is given by the Br-tip low-temperature (LT) STM as shown in Fig. 3c, in which the phenyl rings can be clearly identified. From the zoom-in STM image in Fig. 3d, the formation of a new phenyl group via Bergman cyclization is confirmed (white arrow). To recognize the ethynylene groups, we performed nc-AFM measurement. Figure 3e, f show the Br-tip STM image and nc-AFM frequency shift map of a same area. Similar to many previous reports^26,39–41^, ethynylenes are imaged as bright dots in the nc-AFM frequency shift map (yellow arrow). C 1*s* SRPES shown in Fig. 2a agrees with this conclusion. After the annealing the 315 K sample to 420 K, the signal of C–Ag bond disappears, and only C2 and C3 with BE of 285.3 and 284.6 eV can be observed. The ratio between them is calculated to be 1:3.4, in agreement with the value of 1:3 derived from the molecular model. Note that no signal at BE lower than C3 is observed, implying the radicals generated from C–Br dissociation might be quenched by stray H atoms on the Ag(111) surface (adsorbed from residual gas^29,42–44^). Otherwise, the radical-surface interactions would contribute to a peak at low BE (~283.3 eV)^34^.

### Reaction process

**Fig. 4:**
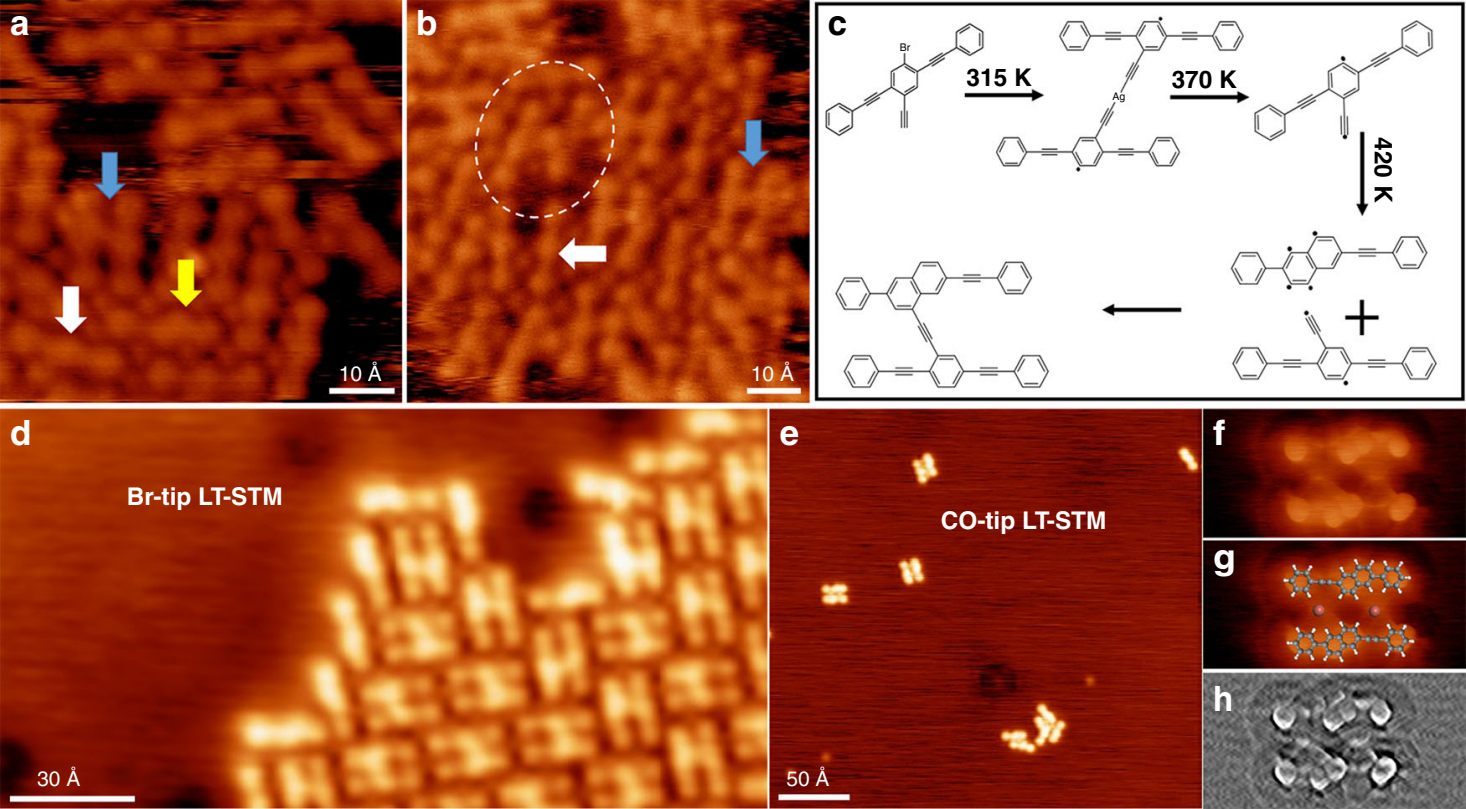
Reaction process and its evidences. **a**, **b** STM images of the sample by annealing to 370 K. Various different intermediate and final products are pointed by different colored arrows, except the white circled organometallic species. **c** The reaction process of the formation of H-type product **4** from molecule **6** on Ag(111). **d** Br-tip LT-STM image of the edge of H-type molecular island. **e** STM image of the sample upon fast annealing from 315 to 420 K. **f**, **g** CO-tip LT-STM image of the non-covalent dimer composed by monomers after cyclization, with and without superimposed molecular models. **h** Laplace and fast Fourier transform (FFT) filtered STM image of the non-covalent dimer in **f**. Tunneling parameters: **a** *V* = −1.5 V, *I* = −0.3 nA; **b** *V* = −2.2 V, *I* = −0.2 nA; **d**, **e** *V* = 0.6 V, *I* = 0.5 nA; **f** *V* = 0.3 V, *I* = 0.5 nA. Color code: C, gray; H, white; Br, red

The combined LT-STM, nc-AFM, and SRPES results shown above consistently confirm the high-yield formation of H-type covalent product **4** on the Ag(111) surface at 420 K. To investigate the reaction process of the formation of **4** from precursor molecule **6**, we annealed the sample prepared at 315 K (0.6 ML) to a relatively low temperature of 370 K to observe the reaction intermediates. It is shown that the surface is covered by disordered structures at these conditions (Fig. 4a, b, overview STM image in Supplementary Fig. 6). The main products of this sample are: (1) monomers (non-cyclization, but involving dehydrogenation and debromination), which are the dominant species (~50%, white arrows); (2) some remaining organometallic structures (~30%), among which a few alkynyl–Ag–phenyl linked species are observed (white dashed circle) besides the alkynyl–Ag–alkynyl structures; (3) H-type final covalent products **4** that start to be generated (~15%, blue arrows); and (4) monomers that have undergone cyclization (~5%, yellow arrow). The experimental observations indicate that the organometallic dimers formed at 315 K are unstable at 370 K. After the release of the interstitial Ag adatoms, the biradical monomers undergo intramolecular Bergman cyclization, and then the generated phenylene radical reacts with a non-cyclization monomer to form the final H-type product **4**. The whole reaction process is shown in Fig. 4c.

Two pieces of experimental evidence support the reaction process as proposed above. First, at the edge of the molecular island of H-type product **4**, some monomers after cyclization can be observed, as shown in Fig. 4d. The asymmetric morphology implies that the Bergman cyclization is involved in their structure. A chemical bond resolved Br-tip LT-STM of a different area is seen in Supplementary Fig. 7, revealing a similar result. At the area with low molecular density and possibly at edges of molecular aggregates (on the 420 K sample), it is relatively difficult for the post-cyclization monomers **2** to find a non-cyclization monomer **1** with which to couple. If the bimolecular cross-coupling between **1** and **2** cannot occur within a short time-scale, more monomers **1** could cyclize to **2**^45–47^, thus a few excess monomers after cyclization are observed. Second, an experiment with fast annealing (~50 K/min) of the 315 K sample to 420 K was performed. About 90% molecules desorb from the surface and almost all of the surviving molecules involve intramolecular Bergman cyclization, but without the formation of H-type product **4**, as shown in Fig. 4e. The majority of these monomers after cyclization assemble into non-covalent dimers. The high-resolution CO-tip LT-STM image of the non-covalent dimers and its Laplace–FFT–filter image are shown in Fig. 4f, g (with superimposed molecular models) and Fig. 4h, respectively. The phenyl ring generated via Bergman cyclization can be clearly distinguished and the two monomers are stabilized via Br⋯H bonds. Two Br adatoms can be identified as two dots between the two monomers. The fast-annealing process gives rise to the low concentration of monomers on the surface due to the strong desorption (~90%); thus, the bimolecular coupling is inhibited, similar to the synthesis of macrocycles under high-dilution conditions^46^. The coupling between two monomers after cyclization is difficult due to the steric hindrance, which will be further discussed below.

### DFT calculation of reaction kinetics

**Fig. 5:**
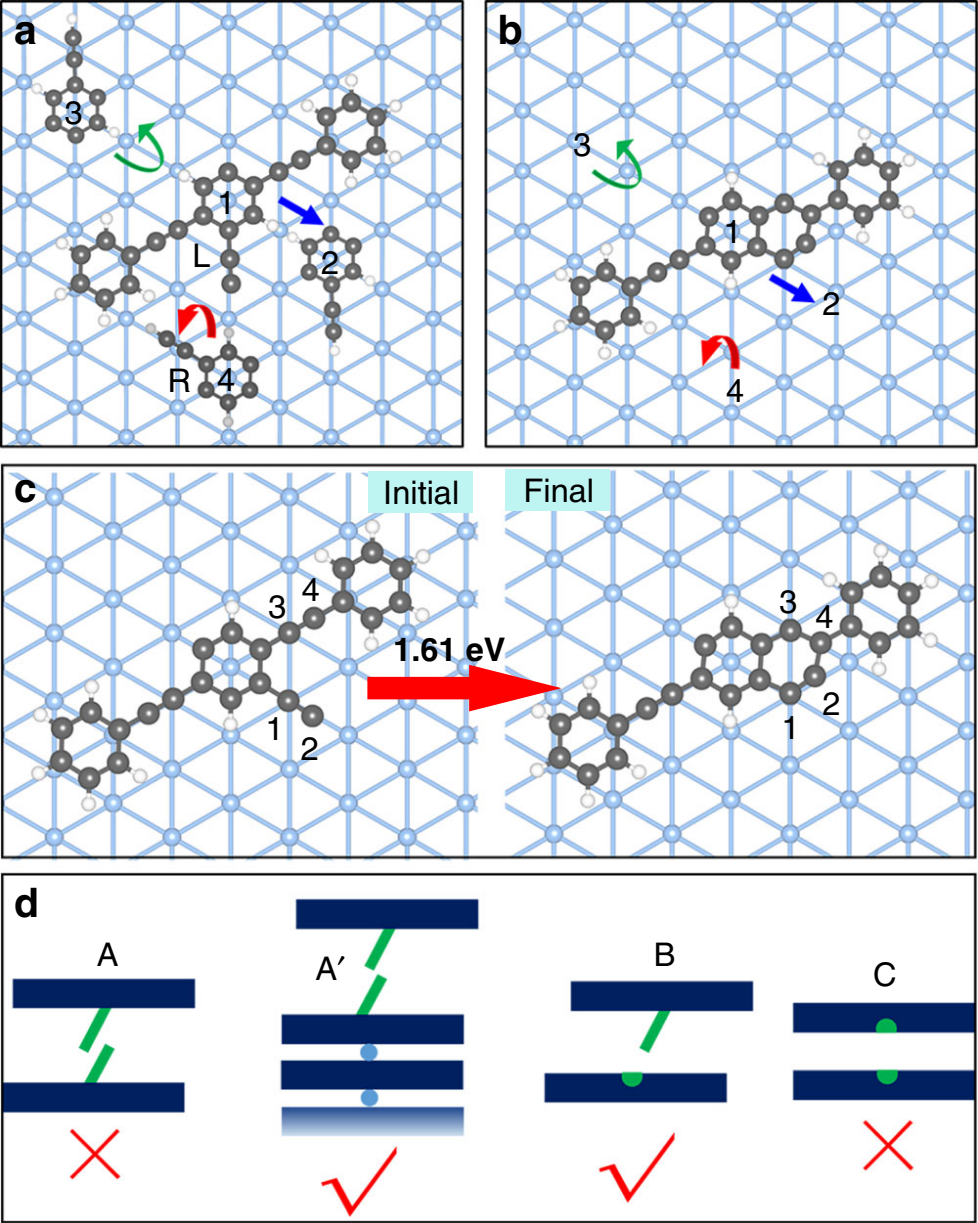
DFT calculations for the molecular motion and intramolecular Bergman cyclization. Schematic illustrations of translation (1 to 2, blue arrow), rotation (1 to 3, green arrow), and inversion (1 to 4, red arrow) for monomers **a** before and **b** after cyclization. For molecule before cyclization, the central ring of the molecule is shown for 2, 3, 4 for clarity of presentation. For molecule after cyclization, only 2, 3, 4 are used for clarity of presentation since it is difficult to find a unit to represent the molecule. **c** Schematic illustration of Bergman cyclization. **d** Models depicting kinetic steric hindrance for different coupling reactions. Dark blue plate represents molecular backbone; green line represents terminal alkynyl; light blue dot represents Ag adatom; green semicircle represents radical in phenylene

It is interesting that high-yield H-type product **4** is generated via heterochiral intermolecular reaction. In this reaction system, the precursor state (370 K sample) for the formation of H-type product **4** shows disordered molecular alignment. Therefore, a prerequisite for this high chiral selectivity is low translation, rotation, and inversion energy barriers for the reactive monomers (in particular, the inversion of the monomers leads to the change of molecular chirality on the surface). In addition, the cyclization process should be the rate-determining reaction step, whereas the molecular motion and the subsequent bimolecular cross-coupling should be relatively fast at the reaction conditions (other bimolecular homo-couplings are inhibited as discussed below). Otherwise, all monomers may undergo intramolecular cyclization. We have investigated three basic molecular motions on Ag(111) through DFT calculations. The motion energy barrier is obtained by scanning the motion pathway from a stable adsorption site (initial state) to the nearest site (final state). For the monomer without cyclization (Fig. 5a), the translation energy barrier is about 0.57 eV (positions 1–2, blue arrow), the rotation energy barrier is 0.61 eV (positions 1–3, green arrow), and the inversion energy barrier is 0.82 eV (positions 1–4, red arrow). For the monomer after cyclization, the three motion energy barriers are calculated to be 0.31, 0.40, and 0.62 eV, respectively (Fig. 5b). The low motion energy barriers of both monomers indicates that the molecular motion is not rate determining and can be considered as free motion above room temperature.

Next, the Bergman cyclization process is investigated, as shown in Fig. 5c. This process is divided into two stages: first, the dehydrogenated terminal alkynyl approaches the alkynyl in the molecular backbone (atom 2 approaching atom 4). At the same time, atom 4 starts to rotate around atom 3 to approach atom 2. The rotations of 2 and 4 atoms result in the transfer of *sp*-like hybridization in the alkynyls into the *sp*^2^-like hybridization of a phenyl ring. Then, atom 2 binds with atom 4 to accomplish the cyclization process. The reaction energy barrier is close to the orbital energy change between *sp*-like and *sp*^2^-like hybridizations, which is about 1.61 eV. Compared to the molecular motion energy barriers, the Bergman cyclization process is expected to be the rate-determining step for the formation of H-type product **4**, consistent with the experimental results.

### Intermolecular coupling

For the bimolecular coupling reaction, it is surprising that no Glaser coupling product between two homochiral molecules is observed. According to previous work, the energy barrier of Glaser coupling between simple linear terminal alkynes is not high (about 1.4–1.5 eV)^48^. After the release of the interstitial metal adatoms (Ag or Au), the covalent coupling can typically be activated below 400 K^48,49^. Therefore, the absence of Glaser coupling products in this system likely originates from steric hindrance between the long molecular backbones of the monomers. This is supported by the fact that Glaser coupling between terminal alkynes with long and stiff molecular backbones is more difficult than simple terminal alkynes^50^, as illustrated in Fig. 5d. The monomer without cyclization is simplified as a long plate (molecular backbone) with a short green line (terminal alkyne). The monomer after cyclization is simplified as a plate including a semicircle (the reactive radical) in its center. In case A, coupling is difficult because of the fast molecular motion of both monomers, that is, the rapid motion after the release of the Ag adatom will make it unlikely to achieve the necessary coupling geometry (crossover of terminal alkynes is more likely^50^, as shown in model A). In this case, the direct coupling becomes impossible and the monomers, driven by the repulsions between the two molecular backbones and between the two terminal alkynyls, would diffuse or flip over to find a better coupling geometry. The difficulty of coupling will be lowered significantly in the case of the reaction between a monomer and an organometallic chain, as shown by A′ (lower possibility to deviate the geometry of coupling since only one monomer are free moving), which is corroborated by a control experiment below. A similar phenomenon was reported previously^36^. In that study, the energy barrier for the dimerization of 1,4-dibromobenzene (nucleation) on Cu(110) is higher than that for the trimerization (further chain growth). For the case B, the coupling of two monomers is relatively easy compared to A because the newly formed ring provides a better geometric factor for reaction with **6**. For the case C, because of the huge steric hindrance between molecular backbones, the coupling of two monomers after cyclization is impossible.

### Template effect of organometallic chain

The results and discussion above demonstrate that the formation of H-type product **4** from reaction between heterochiral molecules **6** on Ag(111) is the kinetically favorable reaction pathway. It is distinctly different from the reaction of molecule **5** on Ag(111), where Glaser coupling prevails. Here, we present a rational argument for the difference in these reaction pathways.

A key difference between the two experiments is that, for the reaction of **5** on Ag(111), the formation of final graphdiyne-type covalent chains undergoes an intermediate state of organometallics^29^, whereas for the reaction of **6** on Ag(111), no organometallic chains are obtained except organometallic dimers. The dissociation of organometallic dimers leads to the generation of biradical monomers and the intramolecular cyclization of these monomers is confirmed to be more kinetically preferred than bimolecular Glaser coupling. These facts imply that the organometallic template plays an essential role for enhancing Glaser coupling, probably because two adjacent molecules in the organometallic chain have a perfect geometry to couple after the release of an interstitial Ag adatom (the organometallic chains are mostly from homochiral molecules; detailed thermodynamic analysis is seen in Supplementary Fig. 8). The relatively weak mobility of the two generated chains guarantees the perfect geometry of the two terminal alkynyls to coupling. In addition, the typical one-by-one dissociation of C–metal bonds in organometallic chains upon annealing maintains a highly efficient templating effect during the growth of covalent chains^29,35^. The mechanism proposed here may be similar to previously reported pre-assembly-directed reaction selectivities (stabilized by hydrogen bonds^51^, metal–organic coordination^52,53^, CH/π interactions^19^). In contrast, for the case of **6** on Ag(111), the high mobility of the two monomers make the Glaser coupling difficult due to the kinetic reasons discussed above, while the single-molecule cyclization could occur leading to the formation of H-type product **4**. In addition, it is noted that the Bergman cyclization in the terminus of a chain should be more difficult compared to the situation of a free-moving monomer due to the constrained motion in the large molecular mass chain. The reaction barrier of the cyclization of the terminal monomer in an organometallic chain is calculated by DFT to be 1.90 eV (Supplementary Fig. 9), much higher than that of free monomer.

**Fig. 6:**
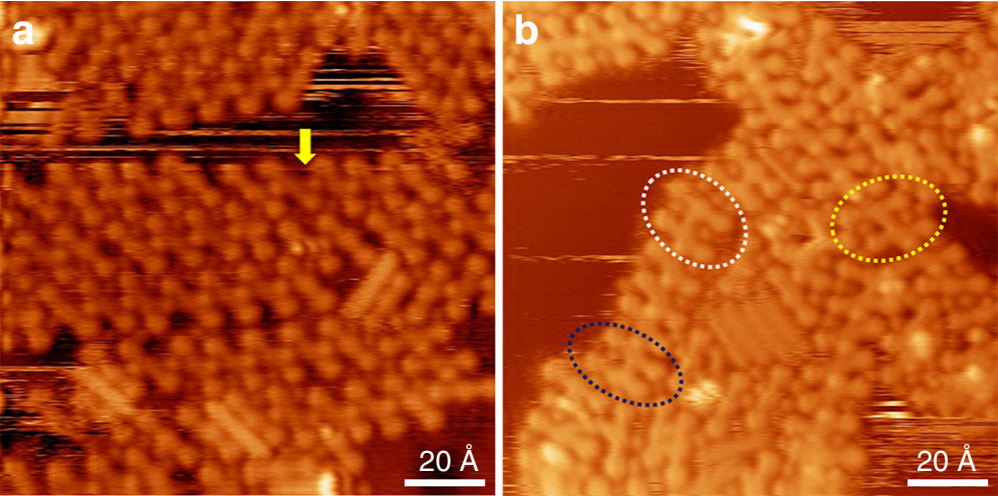
Covalent connection upon annealing of the organometallic chains from **6**. **a** STM image of organometallic chains from **6** at 315 K. One Ag adatom in alkynyl–Ag–alkynyl is pointed by the yellow arrow. **b** STM image recorded after annealing the sample in **a** to 420 K. The H-type product is marked by dark blue circle; Glaser product at the termini of chain is marked by white circle; Glaser product in the middle of chain is marked by yellow circle Tunneling parameters: **a** *V* = −1.6 V, *I* = −0.2 nA; **b** *V* = −1.3 V, *I* = −0.3 nA

To further corroborate the organometallic template effect, a series of control experiments were performed. First, large-area organometallic chains from molecule **6** can be also obtained on Ag(111) by adjusting the annealing procedure. After depositing **6** on Ag(111) at 150 K, slow annealing to 315 K led to stepwise dehydrogenation and debromination. Thus, organometallic chains from homochiral monomers can be obtained, as shown in Fig. 6a (the result is similar from 315K to 370 K, except that chains obtained at 370 K are longer and include phenyl–Ag–phenyl connections; Supplementary Fig. 10). The majority of the connections in the chains are alkynyl–Ag–phenyl with a few alkynyl–Ag–alkynyl (yellow arrow). Figure 6b shows the STM result after annealing the sample in Fig. 6a to 420 K. Some smooth covalent connections can be found on the sample, marked by dashed circles (alkynyl–metal–phenyl bonds are more stable than alkynyl–metal–alkynyl^44,48^; thus, some in chains remain intact at 420 K). For the coupling in the middle of the chains, only Glaser-type coupling is observed, marked as the yellow dashed circle, which is observed as an oblique H shape and has relatively long distance between the two monomers. The center-to-center distance along the molecular backbone is 9.1 ± 0.2 Å, in agreement with previous work^29,48^, and with current DFT calculations. The Glaser-type connection should originate from the release of the Ag adatom in alkynyl–Ag–alkynyl. For the covalent connections at the termini of chains, both H-type and the Glaser coupling product are observed, marked as the dark blue and white dashed circles, respectively. At the chain terminus, as the monomer breaks away, it may undergo intramolecular cyclization and flip over (change its chirality) to react with the alkynyl groups in the residual chain, leading to the formation of H-type product **4**. The non-axis character of the outside monomer of **4** marked by dark blue dashed circle suggests a cyclization step. On the other hand, some Glaser coupling connections can be also observed at the end of the chains (white dashed circle), as expected because the Glaser coupling between a free monomer and a long chain would be easier than that between two free monomers as discussed above (Fig. 5d).

Therefore, a competition is involved between Glaser coupling and the formation of H-type product **4** in the terminus of the chains (Glaser:H≈3:1). These experimental observations corroborate that the template effect of organometallic chains could enhance Glaser coupling, whereas the H-type product **4** can be found only in the terminus of the chains due to the relatively weak template confinement here.

Another control experiment was performed with molecule **5** as the precursor. To avoid the formation of organometallic chains, molecule **5** could be directly deposited on a hot Ag(111) surface. At these conditions, single-molecule cyclization could occur and the H-type covalent product **4** (R1 = alkynyl, R2 = H) is generated. We deposited **5** onto the Ag(111) surface held at 420 K. Although the molecules show strong desorption, many H type covalent products **4** are found in the area close to the step edge (Supplementary Fig. 11). This result suggests that without the organometallic template, **5** can also form H-type covalent products **4** via heterochiral intermolecular reaction. In other words, Bergman cyclization for **5** on Ag(111) is more energetically preferred than Glaser coupling, same as for **6**. Similarly, deposition of **6** onto Ag(111) held at 420 K (weaker desorption) also leads to the formation of high-yield H-type product **4** (R1, R2 = H), accompanied with a few alkynyl–Ag–phenyl linked organometallic chains (Supplementary Fig. 12). For comparison, we repeated the experiment of ref. ^29^. Annealing the alkynyl–Ag–alkynyl linked organometallic chains (prepared by deposition of **5** on Ag(111) held at 330 K) to 420 K demonstrates the prevalence of Glaser coupling (~50%) above other connection types in the covalent chains (Supplementary Fig. 13).

## Discussion

The reaction selectivity of chiral alkynes shown here should provide guidance for the synthesis of graphyne- and graphdiyne-like structures on surfaces. (1) The interesting selectivity of intermolecular cross-coupling as depicted in Fig. 5d may pave a way for enhancing Sonogashira cross-coupling on metal surfaces. Despite its vital importance in the synthesis of graphyne and other *sp*-hybridized low-dimensional nanostructures, the efficient control of Sonogashira coupling has been challenging^15^. The difficulty is mainly due to side reactions, for example, polymerization of alkynyl, Ullman, and Glaser homo-couplings^20^. (2) The protective effect of the metal–alkynyl bond toward the active alkynyl group is similar to that of the silicon- and hydroxyl-based protective groups in wet chemistry^54,55^, which could be utilized for the synthesis of 2D graphdiyne^56,57^, where Bergman cyclization of the *cis*-enediyne unit is one of the most significant side reactions^58^.

**Fig. 7:**
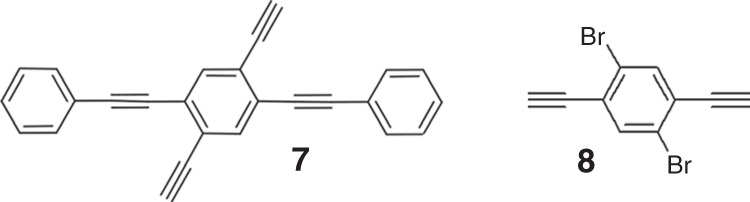
Similar alkyne molecules used in previous studies. The reaction of **7** leads to the formation of graphdiyne covalent nanowire, while the reaction of **8** gives rise to the formation of binodal organometallic network consisting of both alkynyl–Ag–alkynyl and alkynyl–Ag–phenyl links

It is interesting to compare the reactions of structurally similar terminal alkynes on Ag(111) in this and previous works^29,59,60^. Molecule **7** (Fig. 7), which does not contain Br substituents, takes direct alkynyl–alkynyl coupling via a coupling-dehydrogenation mechanism^59^. In contrast, **5**, **6** (Fig. 1), and **8** (Fig. 7) first undergo an alkynyl–Ag–alkynyl organometallic intermediate state where Br adatoms probably promote the single-molecule dehydrogenation^29^. The organometallic species from **5** and **6** transform into covalent structures upon further annealing. However, a gentle annealing of the alkynyl–Ag–alkynyl chain from **8** generates a more thermodynamically stable binodal organometallic network consisting of both alkynyl–Ag–alkynyl and alkynyl–Ag–phenyl links. Thus, one can see that the lattice match between organometallics and the surface contributes significantly to the reactions of alkynes.

We have studied the reaction selectivity of a chiral terminal alkyne on the Ag(111) surface. Using precursor molecules with different substituents, the reaction pathways have been successfully controlled under appropriate kinetic parameters. Homochiral molecule pairs are covalently connected via intermolecular Glaser coupling. Heterochiral cross-coupling between two molecules (one of which involves intramolecular Bergman cyclization) also takes place. It is revealed that the single-molecule Bergman cyclization followed by the formation of H-type product **4** from heterochiral molecules is a kinetically favorable pathway for the reactions of free molecules **5** or **6**. However, the template of the alkynyl–Ag–alkynyl organometallic chain could enhance the Glaser coupling, as shown in the reaction of **5** on Ag(111) in a slow annealing procedure. This present work provides deep insight into different chemical reactions between chiral molecules and offers kinetic strategies to tune reaction pathways.

## Methods

### Experiments

The conventional STM experiments (recorded at 93 K) were performed in a three-chamber UHV system at a background pressure below 2 × 10^–10^ mbar. The scanning tunneling microscope is a SPECS STM 150 Aarhus with SPECS 260 electronics. All voltages refer to the sample and the images were recorded in constant current mode. The Ag(111) single crystal with an alignment of better than 0.1° relative to the nominal orientation was purchased from MaTecK, Germany. Preparation of a clean and structurally well-controlled Ag(111) surface was achieved by cycles of bombardment with Ar^+^ ions and annealing at 700 K, respectively. Molecule **6** with 97% purity was purchased from Alfa Chem, China, and molecule **5** with more than 98% purity was synthesized according to the synthetic route reported in ref. ^29^. The detailed synthetic route for **5** is seen in Supplementary Information. **6** and *Ultra-high vacuum* were vapor deposited from a commercial Kentax evaporator with a Ta crucible held at 408 K for **6** and 433 K for **5**. STM experiments were performed at a sample temperature of 93 K, unless otherwise indicated. The STM images presented here are representative of many images from different regions of the surface obtained in multiple experiments (see image statistics in Supplementary Table 1).

The LT SPM experiments were performed at 4.3 K in a Scienta-Omicron LT-SPM system with a Matrix 3 controller using a qPlus sensor equipped with a tungsten tip (sensor stiffness *k*_0_ ≈1800 N/m, resonance frequency *f*_0_ = 27,118 Hz, and the quality factor *Q* ≈10,000) with a base pressure of 5 × 10^−11^ mbar. The samples were transferred to the LT-SPM system using a UHV suitcase with background pressure below 1 × 10^−9^ mbar. Br-terminated tip was prepared by scanning the Br adatoms on the Ag(111) surface with a bare metal tip, until the Br atom moved onto the tip apex. The CO-terminated tip was prepared by following the routine below. CO at 0.01 ML was deposited on the sample held at 4.3 K first, followed by scanning the CO molecules on the Ag(111) surface with a bare metal tip, until CO moved onto the tip apex^61^.

The SRPES measurements were performed on the Catalysis and Surface Science Endstation located in National Synchrotron Radiation Laboratory (NSRL), Hefei, China. The detailed description of the endstation can be see elsewhere^62^. The SRPES spectra were collected at an emission angle of 45° with respect to the surface normal.

### DFT calculations

All DFT calculations were performed using the Perdew–Burke–Ernzerhof generalized gradient approximation implemented in Vienna ab initio simulation package^63–65^. The plane-wave cutoff energy is set to be 400 eV. The van der Waals interactions were described by D3-Grimme approach^66^. The energy criterion was set to be 10^−5^ eV. The nudge elastic band method was used to estimate the reaction barriers of Bergman cyclization process^67^. The residual forces converging to <0.05 eV/Å.

The Ag(111) surface was modeled with a 10 × 10 × 1 supercell (100 Ag atoms) with coordinates derived from the experimental lattice constant of bulk Ag (4.086 Å)^68^. The supercell was hexagonal with lattice parameters: *a* = *b* = 28.890 Å, *c* = 20 Å, and *γ* = 120°. The vacuum layer along the *z*-axis was set to be above 15 Å and the lateral (*x*, *y*) directions were large enough to avoid the interactions between adsorbates. A few Ag adatoms were added if necessary, to simulate the protrusion phenomenon of surface Ag atoms in organometallic configurations. The supercell shape was fixed, but the Ag atoms of substrates, Ag adatoms, and molecules were fully relaxed until the residual forces were below 0.01 eV/Å. The size of the supercell in the direction perpendicular to the Ag(111) surface is set to 20 Å.

## Supplementary information

Supplementary Information

Peer Review

## Data Availability

The data that support the findings of this study are available from the corresponding authors upon reasonable request.
