# Supplementary material for: Reaction selectivity of homochiral versus heterochiral intermolecular reactions of prochiral terminal alkynes on surfaces
Source: Nat Commun. 2019 Sep 11;10:4122. doi: 10.1038/s41467-019-12102-y (PMC6739358; doi:10.1038/s41467-019-12102-y)
Supplement: Supplementary file 1 — Supplementary Information [file 41467_2019_12102_MOESM1_ESM.pdf]

Supplementary Information for

Reaction selectivity of homochiral versus heterochiral intermolecular reactions of prochiral terminal alkynes on surfaces

Wang *et al.*

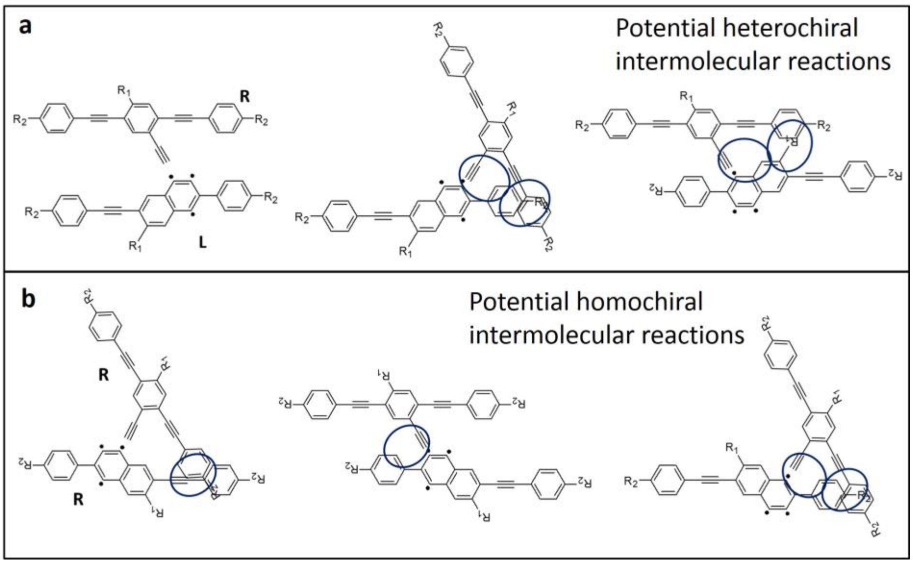

Supplementary Figure 1 All possible **a** heterochiral and **b** homochiral reactions between monomers **1** and **2** (after Bergman cyclization). The first example, also shown in Fig. 1, is the only plausible reaction mechanism and the only one of these that is observed in our experiments. The other cases explore all other possible intermolecular orientations, but none of these can occur because of the huge steric hindrance (marked as blue circles) and none of these are observed in our experiments.

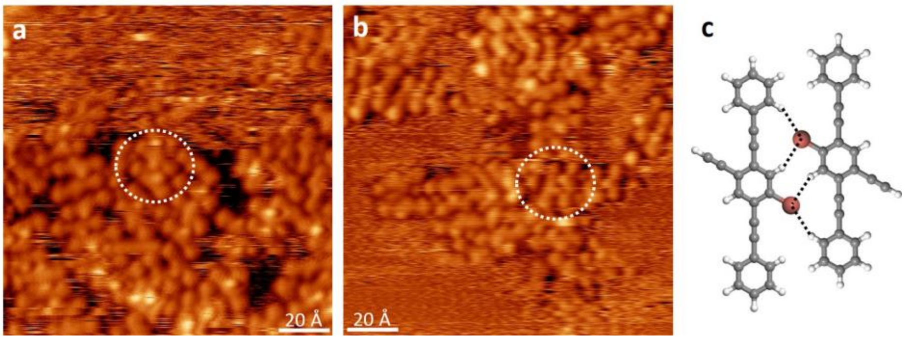

Supplementary Figure 2 STM images of the 150 K sample of molecules **6** with 0.6 ML coverage. **a**, **b** STM images of the sample prepared by deposition of **6** on Ag(111) held at 150 K. **c** Molecular model of the non-covalent dimer as marked by white dashed circles in **a** and **b**. Tunneling parameters: V=3.5 V, I=0.3 nA. Color code: C, grey; H, white; Br, red.

According to the previous works[1,2] and also SRPES shown in Fig. 2, the monomers keep intact for deposition conditions of 150 K surface temperature. They exhibit a rod-like feature and the Br site is identified as a bright protrusion in the STM images. Some non-covalent dimers stabilized by Br···H hydrogen bonds as indicated in Supplementary Fig. 2c can be observed. The fuzzy areas (scanned at ~90 K) in both Supplementary Fig. 2a and 2b imply the high mobility of the molecules on the Ag(111) surface. A few dots between the monomers should be Ag adatoms on the surface, which are trapped by the organic molecules. Similar phenomena have been reported in many previous works[3,4,5].

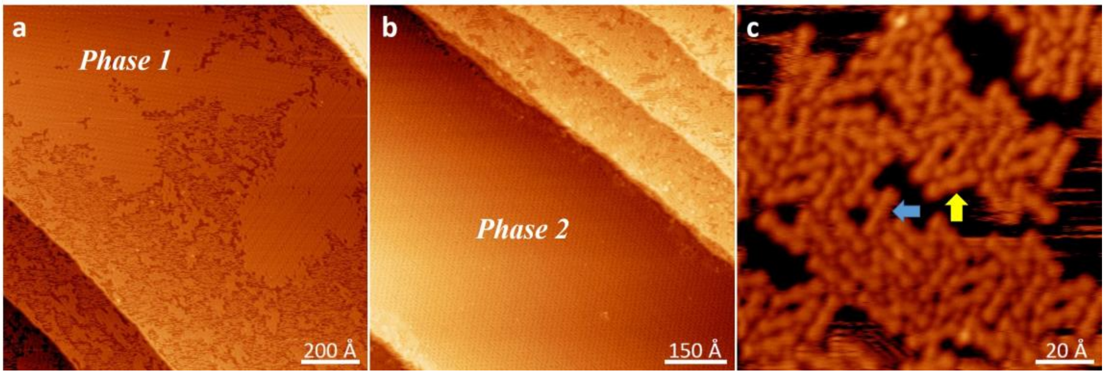

Supplementary Figure 3 STM of 0.6 ML deposition of **6** on Ag(111) with the surface at 315 K sample. **a**, **b** Overview STM images of **a** phase 1 and **b** phase 2 at 315 K. **c** A disordered area at 315 K, showing the coexistence of the two types of organometallic dimer, indicated by blue and yellow arrows. Tunneling parameters: **a** V=−2.5 V, I=−0.2 nA; **b** V= −2.3 V, I=−0.2 nA; **c** V=−1.3 V, I=−0.2 nA.

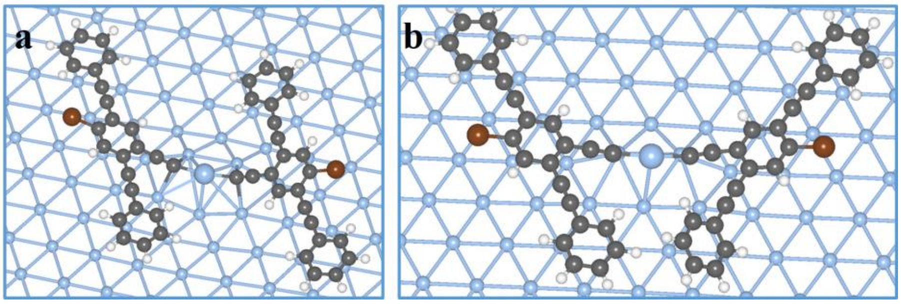

Supplementary Figure 4 Oblique views of the optimized bimolecular organometallic configurations formed at 315 K, starting from molecule **6**. **a** H type dimer 1 from homochiral monomers and **b** A type dimer 2 from heterochiral monomers, corresponding to the top view structures in Figs. 2f and 2g, respectively.

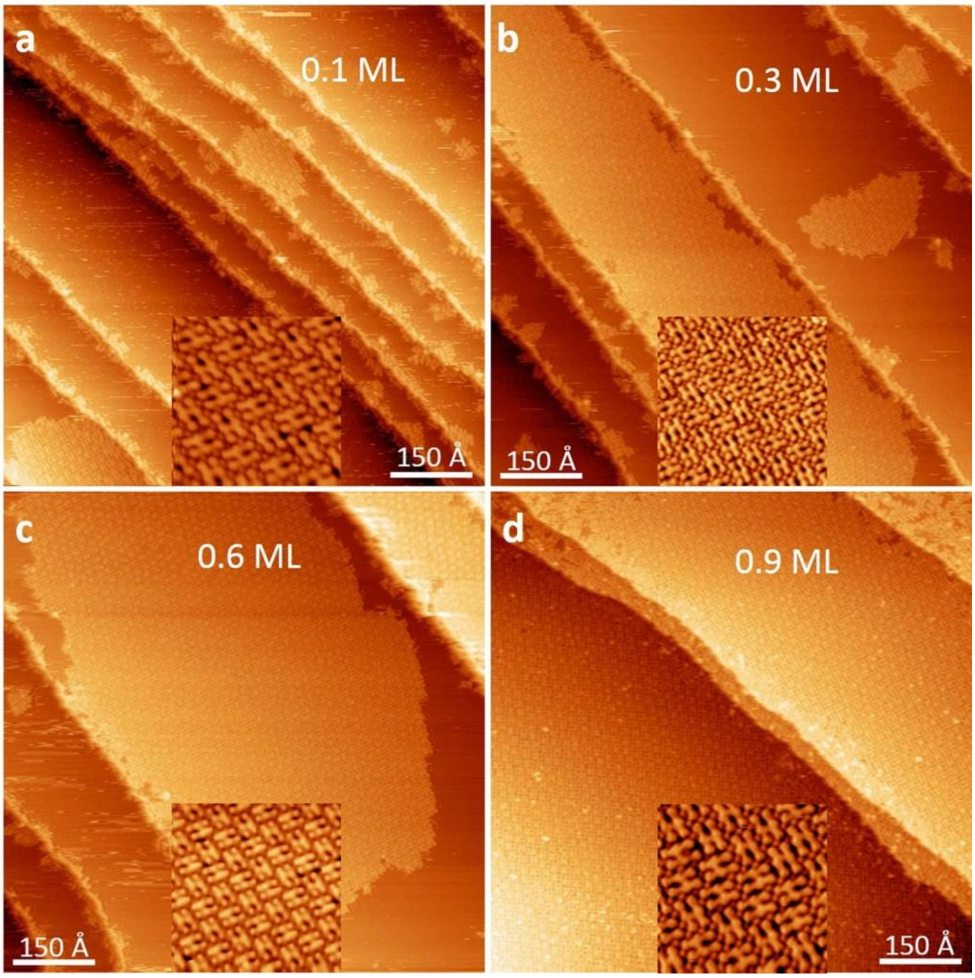

Supplementary Figure 5 STM images of the samples prepared by depositing molecule **6** onto Ag(111) held at 420 K at different coverages, overlaid with the zoom-in STM images of the islands of H product **4** for each. **a** 0.1, **b** 0.3, **c** 0.6, **d** 0.9 ML. Tunneling parameters: **a** V=−2.1 V, I=−0.3 nA; **b** V=−1.7 V, I=−0.2 nA; **c** V=−2.3 V, I=−0.2 nA; **d** V=−2 V, I=−0.2 nA. The large-area close-packed islands of H product **4** dominate at all these samples.

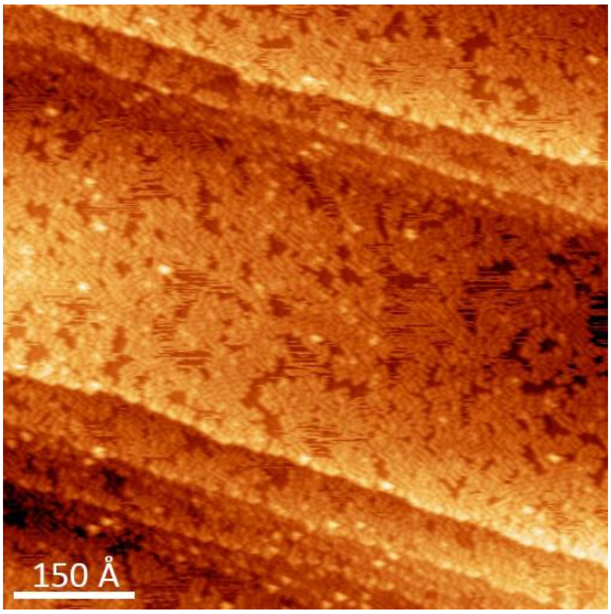

Supplementary Figure 6 Overview STM image of the sample prepared by annealing the 315 K sample (0.6 ML **6**) to 370 K. Tunneling parameters: V=−2 V, I=−0.2 nA.

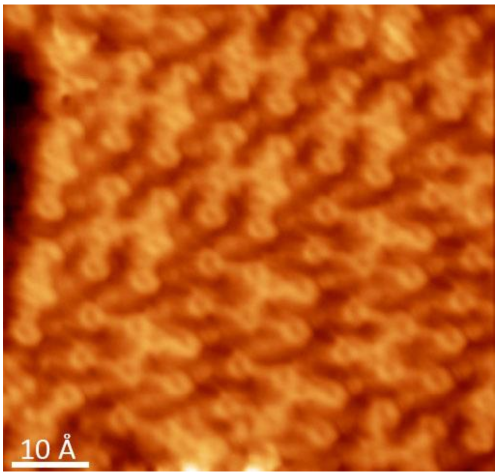

Supplementary Figure 7 Br-tip LT-STM of the sample by depositing 0.6 ML **6** on Ag(111) held at RT , followed by the annealing at 420 K. Some monomers after cyclization can be observed at the edge area. Tunneling parameters: V=0.1 V, I=1.0 nA. $T_{Scanning}$=4.3 K.

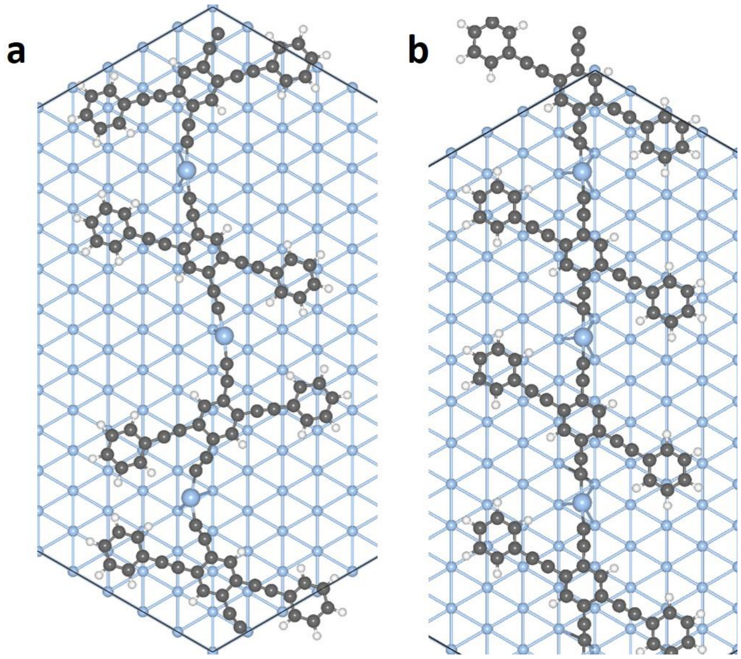

Supplementary Figure 8 The optimized configurations of organometallic chains from molecule **5**. **a** heterochiral and **b** homochiral chain.

To compare the relative stability of homochiral chain vs. heterochiral chain, we have constructed the heterochiral chain and the homochiral chain, as shown in Supplementary Fig. 8. Here, to avoid the interactions between molecules and match the Ag substrate, we have employed 4 molecules and 16×16 supercell of Ag (111) (256 Ag atoms). It shows that the homochiral chain has a low-energy than the heterochiral chain by 0.29 eV per molecule, explaining why the majority of the experimentally observed organometallic chains are homochiral.

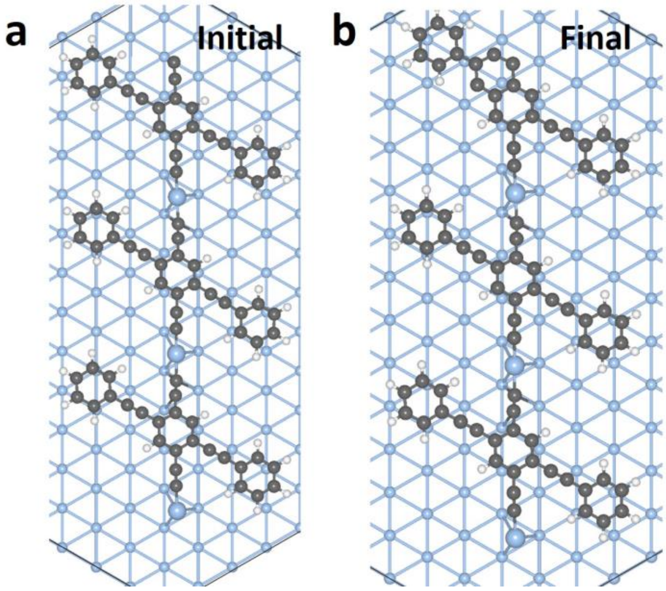

Supplementary Figure 9 DFT energy-minimized structures **a** before and **b** after Bergman cyclization in the terminal molecule (top) of the organometallic chain from molecule **5**. The reaction energy barrier is 1.90 eV.

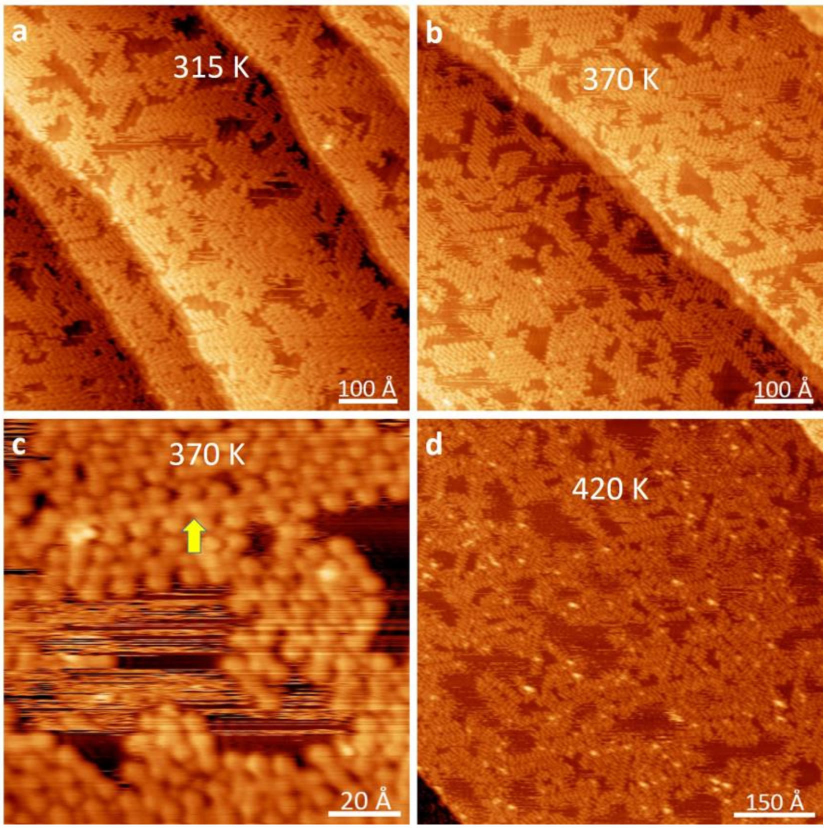

Supplementary Figure 10 Overview STM image of the samples by deposition of 0.6 ML **6** on Ag(111) held at 150 K, followed by the annealing to **a** 315 K, **b, c** 370 K, and **d** 420 K (molecular coverage decreases to 0.4 ML due to thermal desorption). **c** is the zoom-in STM image of an area of **b**. Tunneling parameters: **a** V=−2.5 V, I=−0.2 nA; **b, c** V=−2.1 V, I=−0.2 nA; **d** V=−2.2 V, I=−0.2 nA.

The dehydrogenation and debromination occur slowly at this annealing procedure (the temperature point for the activation of both are possibly very similar). Thus the one-by-one growth mechanism could be involved for the formation of organometallic chains. In contrast, if molecules are directly deposited on Ag(111) held at 315 K, the complete dissociation of C−H and C−Br would be accomplished in a short time. The formation of alkynyl−Ag−alkynyl should be the favorable pathway and these alkynyl−Ag−alkynyl dimers together with some monomers could aggregate into close-packed islands instantaneously (stabilized by Br···H hydrogen bonds) thus inhibiting the growth of organometallic chains. The Br adatoms drive their aggregations. A similar protective effect was reported previously[2].

Different from the case of 315 K, some phenyl−Ag−phenyl connections can be observed at 370 K, as marked by the yellow arrow in Supplementary Fig. 10c. This is most likely attributed to the connection of two chains, which is evidenced by the fact that the lengths of chains at 370 K are overall longer than those at 315 K.

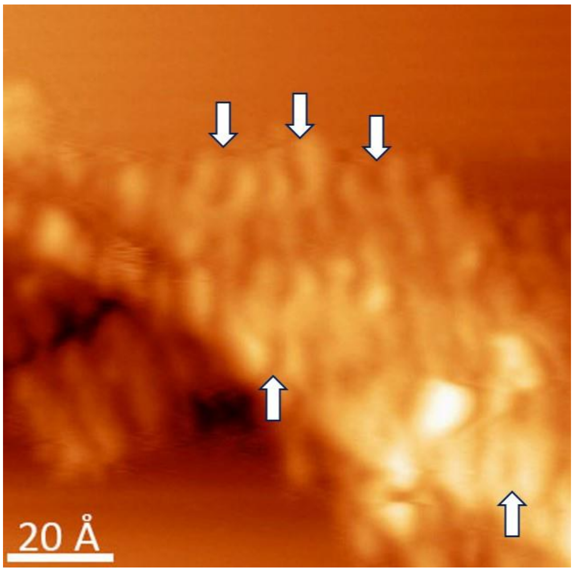

Supplementary Figure 11 STM image of the sample by deposition of **5** onto Ag(111) held at 420 K. The molecule is only adsorbed at step edge areas, with a coverage of ~0.05 ML. Tunneling parameters: V=−2.1 V, I=−0.2 nA.

Although the resolution of the STM image is not very high (STM scan in a step edge area is difficult), the dimeric products should not be connected via Glaser coupling. This is because Glaser coupling is a center-to-center coupling and the center-to-center distance between molecular backbones (9.1 Å in DFT results) should be larger than is observed here (~7 Å). Instead, they are more likely H type products **4** where a Bergman cyclization is involved. This experiment indicates that the Bergman cyclization could occur easily when the template effect of organometallic chain is absent.

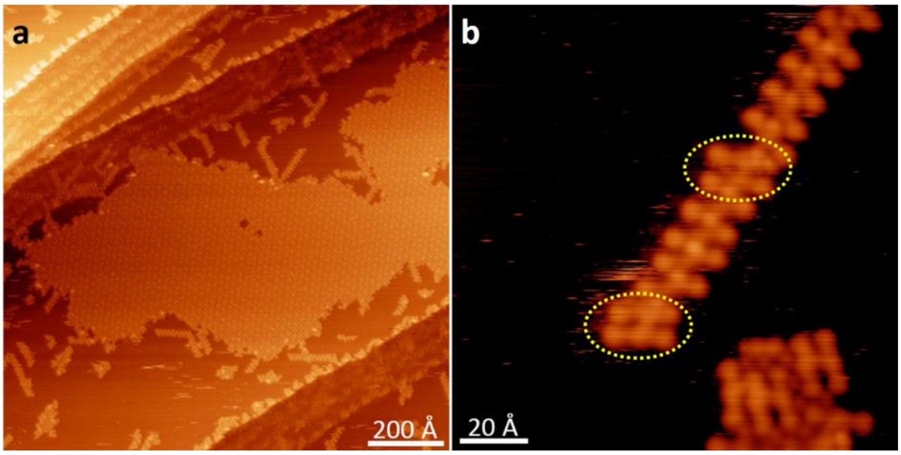

Supplementary Figure 12 STM images of the sample prepared by deposition of 0.5 ML **6** onto Ag(111) held at 420 K. **a** Overview STM image of the sample, where H type product **4** (typically aggregating into close-packed island) is the major products. **b** High-resolution STM image of the organometallic chain, which acts as the minor products on the sample. Tunneling parameters: **a** V=−2.7 V, I=−0.2 nA; **b** V=−2.1 V, I=−0.3 nA.

Because the intramolecular Bergman cyclization (forming **4** in next step) and the formation of organometallic chains manly linked by alkynyl−Ag−phenyl bonds are competitive at a hot surface (alkynyl−Ag−alkynyl is unstable at 420 K), both the two products can be obtained. At the terminal of chains, some H type products **4** are again observed, similar the fact as Fig. 6b shows.

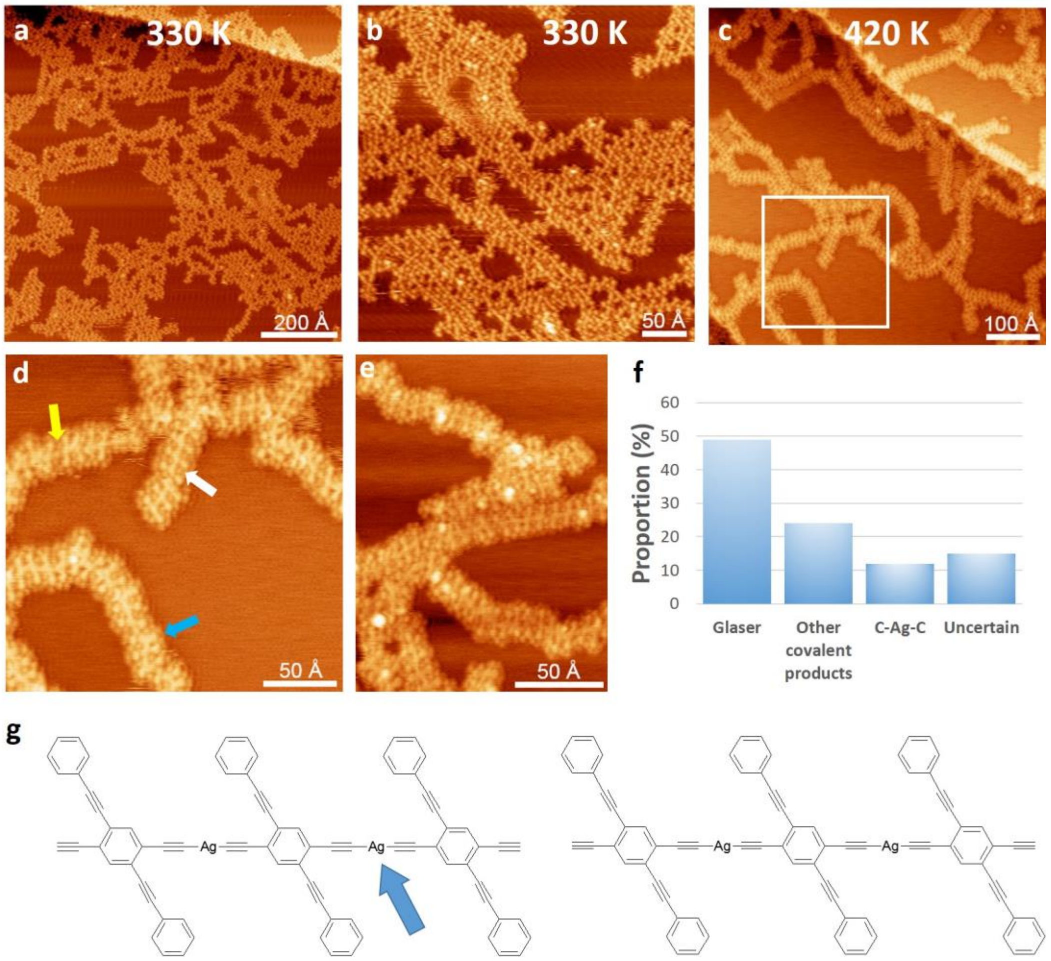

Supplementary Figure 13 STM images of the samples of organometallic and covalent chains from precursor molecule **5**. **a**, **b** Overview and magnified STM images obtained after depositing 0.5 ML **5** onto Ag(111) held at 330 K. **c**, **d**, **e** Typical overview and magnified STM images obtained after annealing the sample in **a** to 420 K (molecular coverage is decreased to 0.3 ML due to thermal desorption). Specifically, **d** is the zoom-in STM image of the white framed region in **c**. **f** Statistic analysis toward different connection types of the chains obtained at 420 K by counting more than 100 monomers. **g** A model for the illustration of the formation of other covalent structures besides the H type product **4**. Tunneling parameters: **a**, **b** V=−1.6 V, I=−0.2 nA; **c**, **d** V=−2 V, I=−0.3 nA; **e** V=−3 V, I=−0.2 nA.

Large-area organometallic chains are obtained upon deposition of **5** on Ag(111) held at 330 K, as shown in Supplementary Fig. 13a and 13b. A 420 K annealing treatment leads to the formation of covalent chains, as exhibited in Supplementary Fig. 13c-e with several connection types. Glaser, other covalent, and C−Ag−C connections are pointed by white, blue, and yellow arrows, respectively (Supplementary Fig. 13c). A statistical analysis toward different connection types of the chains obtained at 420 K (Supplementary Fig. 13f) suggests that Glaser coupling is the main product with a yield of ~50 %, similar as the result in ref. 1. A probable reason for the formation of other covalent connections (involving intramolecular cyclization at most cases) besides Glaser coupling is revealed in Supplementary Fig. 13g. After a release of Ag adatom in a long organometallic chain, two short chains would be formed. In the following step, although the Glaser coupling between the two short

chains could occur, the dissociation of another Ag adatom may be competitive, as pointed by the blue arrow. Once this occurs, the single-molecule cyclization would be involved, leading to the generation of H type product **4** or other covalent products. In addition, for the terminal area of a chain, the formation of H type product **4** can be easier, as discussed in Fig. 6.

	Fig. 2d, 2e, S3	Fig. 3, 4d, S7	Fig. S2	Fig. S5	Fig. 4a, 4b, S6	Fig. 4e	Fig. 6, S10a, S10d	Fig. S10b, c	Fig. S11	Fig. S12	Fig. S13
Experimental times	5	5	3	1	3	2	3	3	2	3	3
Number of Images	62	85	38	15	39	12	40	28	19	42	48
Size (Å) (average)	300×300	200×200	150×150	500×500	300×300	200×200	300×300	300×300	100×100	300×300	200×200

Supplementary Table 1. A summary of the number of experiments, STM images, and sizes of STM images recorded at each set of experimental parameters reported in this work. The number of experiments refers to the number of times that the same experimental conditions were repeated on different samples. The total number of images recorded in those experiments is recorded in the same row. In each case, many images have been recorded in different areas of the sample and on multiple samples; representative images are presented in the manuscript and SI.

Synthetic procedure for molecule **5**.

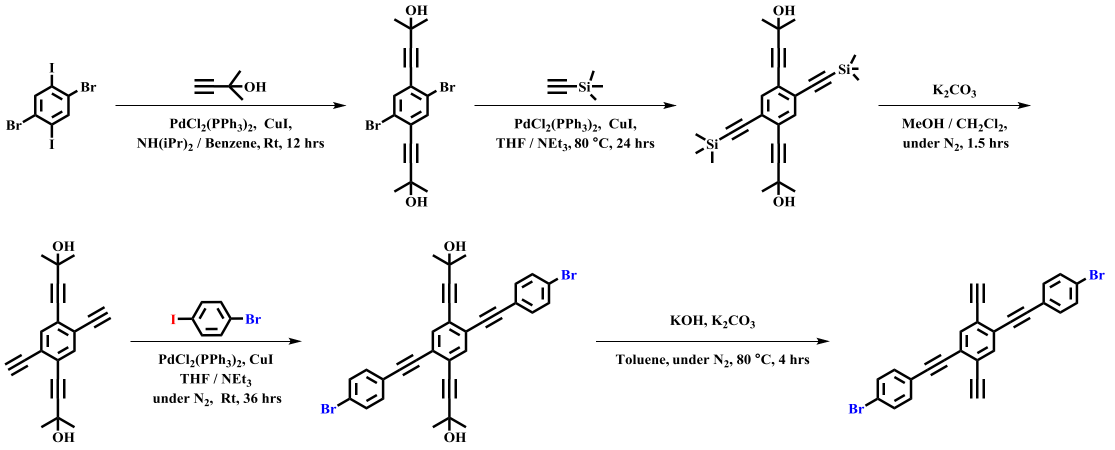

Supplementary Reference

1. Liu, J. *et al.* Bromine adatom promoted C–H bond activation in terminal alkynes at room temperature on Ag (111). *Phys. Chem. Chem. Phys.* **20**, 11081-11088 (2018).
2. Wang, T. *et al.* Highly selective synthesis of *cis*−enediynes on a Ag (111) surface. *Angew. Chem. Int. Ed.* **56**, 4762-4766 (2017).

3. Fan, Q. *et al.* Surface adatom mediated structural transformation in bromoarene monolayers: precursor phases in surface Ullmann reaction. *ACS Nano* **12**, 2267-2274 (2018).
4. Huang, H. *et al.* Competition between hexagonal and tetragonal hexabromobenzene packing on Au(111). *ACS Nano* **10**, 3198-3205 (2016).
5. Lewis, E. A., Murphy, C. J., Liriano, M. L. & Sykes, E. C. H. Atomic-scale insight into the formation, mobility and reaction of Ullmann coupling intermediates. *Chem. Commun.* **50**, 1006-1008 (2014).
