# Supplementary material for: Reaction selectivity of homochiral versus heterochiral intermolecular reactions of prochiral terminal alkynes on surfaces
Source: Nat Commun. 2019 Sep 11;10:4122. doi: 10.1038/s41467-019-12102-y (PMC6739358; doi:10.1038/s41467-019-12102-y)
Supplement: Supplementary file 2 — Peer Review [file 41467_2019_12102_MOESM2_ESM.pdf]

Reviewers' comments:

Reviewer #1 (Remarks to the Author):

In an extensive set of reactions, supported by DFT caluclations, these authors report on controlling selectivity between homochiral and heterochiral reactoin pathwas on surfaces. This study addresses competing reactions of a prochiral alkyne on Ag(111) under HUV conditions, namely homochiral Glaser coupling and heterochiral cross-coupling with a Bergman cyclization step. Tuning the precursor substitutents and the kinetic parameters, control strategies were developed to steer the reaction betwen the so-called homochiral and heterochiral pathways. High-resolution scanning probe microscopy, including both STM and ncAFM, are used. A clear difference if found between the reaction of organometallic chains that enhances Glaser coupling versus the reaction of free monomers, where het kinetically favorable reaction pathway is the cross-coupling between two heterochiral molecules. One of these molecules involves cyclization.

This work is a relevant contribution in the area of on-surface chemistry, though the practical value is probably somewhat limited. Nevertheless, such studies are important to reveal the aspects at play in controlling reactions on surfaces, in particular those that involve several possible reaction outcomes. The system presented is a particular complex one, involving several possible reaction outcomes. Parallels are drawn with the analogous reactions in wet chemistry.

The paper is well written, and the structure is logical. I don't have comments on the scientific approach.

I question though the validity of using the terminology of "chiral" and "homochiral" in the title, and in the text. Also the introduction on chirality seems not very relevant, for the simple reason that in this study it is not possible to control the chirality. The molecules are prochiral. Without chiral bias, on the macroscale, the monomers and reaction products are racemic. I would recommend the authors to tone down their claims. The general readership will expect something else than "2D chirality".

Argumentation on the statistics and reproducibility could have been developed in a more extensive fashion. Using SPM as local probe technique, only a small fraction of the surface is probed. Though I have no doubts that the authors performed their experiments in a decent fashion, I expect detailed information on the "statistics" (e.g. total area probed, number or images, sessions, etc.)

Reviewer #2 (Remarks to the Author):

In this work Wang and coworkers present the on-surface reaction of chiral di-alkyne precursors on Ag(111) surface. Different reactions steps are detailed to explore the reaction mechanisms. It is shown that good control can be achieved on the reaction selectivity by properly adjusting the reaction conditions and/or the precursor design. The experimental work is done by low-temperature STM/AFM and photoemission spectroscopy, and supported by DFT calculations. The work is of high quality and the system is analyzed in depth and in convincing manner. The achievement of selectivity control remains a major challenge in on-surface synthesis field and the manuscript is therefore perfectly timely. The presented work is particularly insightful for the on-surface synthesis community.
The claim for novelty is however less convincing. It is rather difficult to understand what is the main message of the manuscript with regards to novelty. In particular, the molecular family used (type 1) has been previously proposed and it is necessary to summarize these previous results and to put them in perspective with the presented work. See e.g. ref. 32; Liu, J., et al. (2015). Acs Nano 9(6): 6305-6314; Liu, Jing; Chen, Qiwei; Cai, Kang; et al., NATURE COMMUNICATIONS, 10, 2545 (2019).

Minor points :

- It would be interesting to mention the surface densities of the two phases in Fig.2, but also probably for all the systems?!
- indicate which molecule (5 or 6) is used for all figures in SI.

Reply to the reviewers for Manuscript #: NCOMMS-19-19323.

Response to the reviewers: We appreciate the reviewers' helpful comments and suggestions, which are shown below (in italics) together with our detailed responses (in normal font and color blue). **Corresponding revisions in the manuscript and supplementary information are highlighted.**

Reviewer 1:

Reviewer #1 (Remarks to the Author):

In an extensive set of reactions, supported by DFT caluclations, these authors report on controlling selectivity between homochiral and heterochiral reactoin pathwas on surfaces. This study addresses competing reactions of a prochiral alkyne on Ag(111) under HUV conditions, namely homochiral Glaser coupling and heterochiral cross-coupling with a Bergman cyclization step. Tuning the precursor substitutents and the kinetic parameters, control strategies were developed to steer the reaction betwen the so-called homochiral and heterochiral pathways. High-resolution scanning probe microscopy, including both STM and ncAFM, are used. A clear difference if found between the reaction of organometallic chains that enhances Glaser coupling versus the reaction of free monomers, where het kinetically favorable reaction pathway is the cross-coupling between two heterochiral molecules. One of these molecules involves cyclization.

This work is a relevant contribution in the area of on-surface chemistry, though the practical value is probably somewhat limited. Nevertheless, such studies are important to reveal the aspects at play in controlling reactions on surfaces, in particular those that involve several possible reaction outcomes. The system presented is a particular complex one, involving several possible reaction outcomes. Parallels are drawn with the analogous reactions in wet chemistry.

The paper is well written, and the structure is logical. I don't have comments on the scientific approach.

Response: We appreciate the reviewer's accurate evaluation and very positive comments to our work.

I question though the validity of using the terminology of "chiral" and "homochiral" in the title, and in the text. Also the introduction on chirality seems not very relevant, for the simple reason that in this study it is not possible to control the chirality. The molecules are prochiral. Without chiral bias, on the macroscale, the monomers and reaction products are racemic. I would recommend the authors to tone down their claims. The general readership will expect something else than "2D chirality".

Response: We thank the review for this great point and giving important suggestions.

According to the suggestion of the reviewer, we have toned down the claims such as “chiral”, “homochiral/heterochiral” into “prochiral” and “two-dimension homochiral/heterochiral‘’ *etc* in the revised manuscript (including title, abstract, introduction).

1) The title has been changed into “**Reaction selectivity of prochiral terminal alkynes: homochiral versus heterochiral intermolecular reactions on surfaces**” from the original “*Reaction selectivity of chiral terminal alkynes on surfaces: homochiral versus heterochiral intermolecular reactions*”.

2) The second sentence of the abstract section has been revised as “**Here, competing reactions of a prochiral alkyne on Ag(111): two-dimensional (2D) homochiral Glaser coupling and heterochiral cross-coupling with a Bergman cyclization step have been examined.**” from the original “*Here, competing reactions of a chiral alkyne on Ag(111): homochiral Glaser coupling and heterochiral cross-coupling with a Bergman cyclization step have been examined.*”. We state that the chirality of prochiral alkyne **1** is induced by the surface confinement in the introduction section, thus “homochiral/heterochiral” are used in the following text.

3) The introduction on chirality at the beginning of the introduction section is modified in the revised manuscript by deleting irrelevant content. The original statement “*A molecular structure is chiral if it cannot be superimposed onto its mirror image. Owing to the homochiral nature of the biochemistry of life on the earth, molecular chirality has been one of the most important research topics in biology and pharmaceutics for over half a century*[1]. *Although enantiomers of chiral molecules often exhibit the same physical and chemical properties, significant differences emerge when they interact with the homochiral chemical units of biological systems, such as proteins, and poly-saccharides*[2]. *Different enantiomers usually have different effects on biological systems because the interactions between homochiral pairs are very different from those between heterochiral pairs*[3,4]*.*” has been shorten to be “**A molecular structure is chiral if it cannot be superimposed onto its mirror image. Owing to the homochiral nature of the biochemistry of life on the earth, molecular chirality continues to be one of the most important research topics in biology and chemistry.**[1]”

Argumentation on the statistics and reproducibility could have been developed in a more extensive fashion. Using SPM as local probe technique, only a small fraction of the surface is probed. Though I have no doubts that the authors performed their experiments in a decent fashion, I expect detailed information on the “statistics” (e.g. total area probed, number or images, sessions, etc.)

Response: Very good suggestion! We added statistics about the number of images and the image sizes in supplementary information (**Table S1**), which is also stated in method section. The STM images reported in this work are representative of many images recorded in different areas of the surface and over multiple repeat experiments.

Reviewer #2 (Remarks to the Author):

In this work Wang and coworkers present the on-surface reaction of chiral di-alkyne precursors on Ag(111) surface. Different reactions steps are detailed to explore the reaction mechanisms. It is shown that good control can be achieved on the reaction selectivity by properly adjusting the reaction conditions and/or the precursor design. The experimental work is done by low-temperature STM/AFM and photoemission spectroscopy, and supported by DFT calculations. The work is of high quality and the system is analyzed in depth and in convincing manner. The achievement of selectivity control remains a major challenge in on-surface synthesis field and the manuscript is therefore perfectly timely. The presented work is particularly insightful for the on-surface synthesis community.

Response: We really appreciate the reviewer's very positive comments to our work.

The claim for novelty is however less convincing. It is rather difficult to understand what is the main message of the manuscript with regards to novelty. In particular, the molecular family used (type 1) has been previously proposed and it is necessary to summarize these previous results and to put them in perspective with the presented work. See e.g. ref. 32; Liu, J., et al. (2015). Acs Nano 9(6): 6305-6314; Liu, Jing; Chen, Qiwei; Cai, Kang; et al., NATURE COMMUNICATIONS, 10, 2545 (2019).

Response: The reviewer brings a good point to improve our manuscript. First, the introduction on chirality has been made much more concise, as also shown in the response to Reviewer #1, to highlight the novelty of "selective control of homochiral vs. heterochiral intermolecular reaction pathways on surfaces." Second, the reviewer gives three very good examples which could be used to compare with our work. As suggested, we added a figure (Fig. 7) and a short summary of these works in the discussion section. It reads:" **It is interesting to compare the reactions of structurally similar terminal alkynes on Ag(111) in this and previous works[29,59-60]. Molecule 7 (Fig. 7), which does not contain Br substituents, takes direct alkynyl−alkynyl coupling via a coupling-dehydrogenation mechanism[59]. In contrast, 5, 6 (Fig. 1), and 8 (Fig. 7) first undergo an alkynyl−Ag−alkynyl organometallic intermediate state where Br adatoms probably promote the single-molecule dehydrogenation[29]. The organometallic species from 5 and 6 transform into covalent structures upon further annealing. However, a gentle annealing of the alkynyl−Ag−alkynyl chain from 8 generates a more thermodynamically stable binodal organometallic network consisting of both alkynyl−Ag−alkynyl and alkynyl−Ag−phenyl links. Thus, one can see that the lattice match between organometallics and the surface contributes significantly to the reactions of alkynes.**"

Minor points :

- It would be interesting to mention the surface densities of the two phases in Fig.2, but also probably for all the systems?!

Response: Good suggestion! The molecular coverages of all the systems have been mentioned in the revised manuscript and supplementary information, as highlighted in either figure captions or texts. In addition, the ratio between phase 1 and phase 2 of the 315 K sample (Fig.2 and Fig. S3), and the yields of all the intermediates of 370 K sample (Fig. 4) are also indicated. They read: "**two types of well-ordered self-assembly phases are formed (each of them accounts for 40% of organic adsorbates), as shown in Figs. 2d and 2e (overview STM images and disordered phases are shown in Supplementary Fig. 3).**" ; "**The main products of this sample are: (1) monomers (non-cyclization, but involving dehydrogenation and debromination), which are the dominant species (~50%, white arrows); (2) some remaining organometallic structures (~30%), among which a few alkynyl−Ag−phenyl linked species are observed (white dashed circle) besides the alkynyl−Ag−alkynyl structures; (3) "H" type final covalent products 4 that start to be generated (~15%, blue arrows); and (4) monomers that have undergone cyclization (~5%, yellow arrow).**".

- *indicate which molecule (5 or 6) is used for all figures in SI.*

Response: We thank the reviewer for his/her helpful suggestion. We have indicated the corresponding molecule (**5** or **6**) in all figures in the revised supplementary information to make the statement clearer.

REVIEWERS' COMMENTS:

Reviewer #1 (Remarks to the Author):

I feel that all the concerns have been addressed in an appropriate way. I support acceptance.

Reviewer #2 (Remarks to the Author):

I am sorry for the misunderstanding, my suggestion was certainly ambiguously expressed. I was in fact suggesting to provide an estimation of the molecular density for each phase, i.e. the inverse of the surface area of their respective unit cell (how many molecule/precursor per nm^2). This is an important parameter that tends to be maximized at equilibrium and thus could indicate that other effects are taking place in intermediate cases.
Otherwise the authors have responded satisfactorily to the few additional comments from both reviewers.

Reply to the reviewers for Manuscript #: NCOMMS-19-19323A.

Response to the reviewers: We appreciate the reviewers' positive comments, which are shown below (in italics) together with our detailed responses (in normal font and color blue).

Reviewer 1:

Reviewer #1 (Remarks to the Author):

I feel that all the concerns have been addressed in an appropriate way. I support acceptance.

Response: We thank the reviewer for his/her positive comments to our responses and acceptance of our work.

Reviewer 2:

Reviewer #2 (Remarks to the Author):

I am sorry for the misunderstanding, my suggestion was certainly ambiguously expressed. I was in fact suggesting to provide an estimation of the molecular density for each phase, i.e. the inverse of the surface area of their respective unit cell (how many molecule/precursor per nm^2). This is an important parameter that tends to be maximized at equilibrium and thus could indicate that other effects are taking place in intermediate cases.
Otherwise the authors have responded satisfactorily to the few additional comments from both reviewers.

Response: We thank the reviewer's great suggestion. The molecular density for each phase in Fig.2 is included in the revised manuscript. It reads: "**The molecular densities are calculated to be 0.60 monomer per nm^2 for phase 1 and 0.64 monomer per nm^2 for phase 2, respectively.**"
